# Proteomic, metabolomic and lipidomic profiles in community acquired pneumonia for differentiating viral and bacterial infections

**DOI:** 10.1038/s41598-025-85229-2

**Published:** 2025-01-14

**Authors:** Samuel Rischke, Robert Gurke, Ann-Sophie Zielbauer, Nicole Ziegler, Lisa Hahnefeld, Michaela Köhm, Aimo Kannt, Maria JGT Vehreschild, Gerd Geisslinger, Gernot Rohde, Carla Bellinghausen, Frank Behrens, CAPNETZ Study group

**Affiliations:** 1 Institute of Clinical Pharmacology, Faculty of Medicine, Goethe University Frankfurt, Theodor Stern-Kai 7, 60590 Frankfurt am Main, Germany; 2 Fraunhofer Institute for Translational Medicine and Pharmacology (ITMP), Theodor-Stern-Kai 7, 60596 Frankfurt am Main, Germany; 3 Fraunhofer Cluster of Excellence for Immune Mediated Diseases (CIMD), Theodor-Stern-Kai 7, 60596 Frankfurt am Main, Germany; 4 Department of Internal Medicine, Infectious Diseases, University Hospital, Goethe University Frankfurt, Theodor-Stern-Kai 7, 60590 Frankfurt am Main, Germany; 5 Division of Rheumatology, University Hospital, Goethe University Frankfurt, Theodor-Stern- Kai 7, 60590 Frankfurt am Main, Germany; 6 Department of Respiratory Medicine, Medical Clinic I, University Hospital, Goethe University Frankfurt, Theodor-Stern-Kai 7, 60590 Frankfurt/Main, Germany; 7 CAPNETZ STIFTUNG, Carl-Neuberg-Str. 1, 30625 Hannover, Germany; 8 Biomedical Research in Endstage and Obstructive Lung Disease Hannover (BREATH), German Center for Lung Research (DZL), Carl-Neuberg-Str. 1, 30625 Hannover, Germany

**Keywords:** Bacterial infection, Viral infection, Diagnostic markers

## Abstract

Community-acquired pneumonia (CAP) has a significant impact on public health, especially in light of the recent SARS-CoV-2 pandemic. To enhance disease characterization and improve understanding of the underlying mechanisms, a comprehensive analysis of the plasma lipidome, metabolome and proteome was conducted in patients with viral and bacterial CAP infections, including those induced by SARS-CoV-2. Lipidomic, metabolomic and proteomic profiling were conducted on plasma samples of 69 patients suffering either from viral or bacterial CAP. Lipid and metabolite analyses were LC-MS-based, while proteomic analyses were performed using multiple panels of the Olink platform. Statistical methods, machine learning and pathway analyses were conducted investigating differences between the infection types. Through comparison of the bacterial and viral pathogen groups, distinct signatures were observed in the plasma profiles. Notably, linoleic acid-derived inflammation signaling metabolites (EpOME and DiHOME) were increased in viral CAP compared to bacterial CAP. Similarly, proteins involved in cellular immune response and apoptosis (LAG-3 and TRAIL) showed elevated levels in viral CAP, while bacterial CAP exhibited notable elevation in pattern-recognizing receptors (CLEC4D and EN-RAGE). Additionally, within the lipidomic profile at baseline, several lipids displayed notable differences between viral and bacterial pneumonia, including bile acids (GCA, TCA, TCDCA), various tri- and diglycerides (TGs and DGs), and several phosphatidylcholines (PCs). These findings hold promise for facilitating the differential diagnosis of viral and bacterial pulmonary infections based on the systemic lipidome, metabolome and proteome, enabling timely treatment decisions. Additionally, they highlight potential targets for drug research, advancing therapeutic interventions in CAP. By providing valuable insights into the molecular characterization of CAP, this study contributes to the improvement of understanding the disease and, ultimately, the development of effective treatment strategies.

## Introduction

Despite intensive medical research and overall improvement in medical science and public health, pneumonia remains a significant health risk and burden for the healthcare system^1^which was just recently impressively demonstrated by the Covid-19 pandemic^2,3^. The community-acquired pneumonia (CAP) is the most prevalent form of pneumonia, yet CAP is still an often-underestimated healthcare threat^4^ with a notably high lethality^5^. A recently published review by Pletz et al. highlighted the utmost importance of biomarker research and risk stratification in paving the way for an improved CAP treatment in the future^3^.

Biomarkers are quantifiable markers that can indicate normal biological processes, disease-related processes, or biological responses to treatment. They have diverse applications, serving as diagnostic tools, markers of disease activity and severity, predictors of treatment outcomes, and indicators of disease progression. The Federal Drug Administration – National Institute of Health (FDA-NIH) Biomarker Working Group has defined seven distinct categories of biomarkers: diagnostic, risk, prognostic, predictive, monitoring, response, and safety markers^6^. In the context of CAP, biomarkers could be useful tools for early identification of patients with a severe infection and for early differentiation between viral and bacterial infection. Especially considering number of multi-resistant bacteria, this could reduce avoidable antibiotic therapies in viral CAP. Moreover, the utilization of predictive or monitoring biomarkers would significantly enhance the effectiveness of patient treatment^3^. Blood-based omics profiling can be expected to be a valid route for the distinction of causative pathogens^7,8^. While traditional microbiological techniques are likely to be relevant in the future as well, high-throughput methods offer approachable alternatives for rapid phenotyping^9^. Endogenous substances, like procalcitonine, which has been proposed for the distinction of viral and bacterial CAP, demonstrate that, despite being insightful, they need to be evaluated in context of other systemic factors^8,10^.

The identification of reliable biomarkers for respiratory infections, including CAP, is complicated by the complex interplay of host factors, pathogen characteristics, and the diverse immune responses encountered in individuals. This necessitates careful consideration of the specific pathogen type (viral or bacterial) when conducting biomarker research^3^. Given the highly diverse patient population in CAP, single omics studies alone may not be sufficient for identifying robust biomarkers. While numerous single omics studies have demonstrated the potential to identify biomarkers^11–15^, the integration of different omics approaches holds the potential to provide a more comprehensive and promising strategy for biomarker discovery and to improve the understanding of pathophysiological processes in CAP^16–18^. This approach adds further biological context to observations made within single omics domains^19^. Recent work pioneered the connected analysis of metabolomics and proteomics in CAP, tackling the differentiation of causative pathogens in viral infections, or the prediction of disease severity based on blood samples^20,21^. These studies demonstrate the successful application of multi-omics technologies in tackling frontiers in CAP.

In the present work, an omics approach combining the analysis of the plasma lipidome, metabolome and proteome of 69 patients with either viral or bacterial CAP was applied, and the resulting data were compared. To gain a comprehensive understanding of the unique inflammatory pathways, potential signatures for viral and bacterial CAP were analyzed. This integrated approach bears the potential for a better understanding of differentiating factors between infections with different pathogens and will ultimately be of high importance for enabling their individualized therapy^14^. Specifically, the study focused on investigating whether there are discernible differences between viral CAP (including SARS-CoV-2) and CAP caused by bacterial pathogens. Samples for the study were acquired from the CAPNETZ biobank and were analyzed by high-throughput immuno-based assays (Proteomics) as well as LC-MS based methods (Lipidomics and Metabolomics).

## Methods

### Sample collection and study design

Human plasma samples from 69 individuals with either bacterial (*N* = 26) or viral (*N* = 43) respiratory infection were obtained from the CAPNETZ biobank. Sample collection of the present cohort was performed in 17 German hospitals or HIV centers between the 3rd of April 2017 and the 9th of July 2020, with 57% of samples being obtained in 2020. Patients were either in outpatient treatment or hospitalized. All samples were processed for storage in the central biobank using standardized, cross-center standard operating procedures (SOPs) and trained personnel to ensure consistency and reliability in sample handling and quality. All subjects included in the CAPNETZ study were > 18 years old and were consenting to study participation voluntarily. The study was conducted adhering to the Declaration of Helsinki, as well as guidelines of good clinical practice (GCP) and good epidemiological practice (GEP). It was approved by the institutional ethics board of the Hannover Medical School (Ethics approval No. 301–2008) and the responsible local review boards of the participating centers. Further inclusion criteria included evidence of lung infiltrate by imaging, and at least one of the following: active coughing, purulent sputum, positive auscultation findings, or fever. It should be noted that a minor number of COVID-19 patients did not exhibit lung infiltrates; therefore, by definition, they were not classified as having CAP. However, these patients were still included in the study due to the severity of their illness and were subsequently analyzed together with the other viral CAP patients in one group. Exclusion criteria on the other hand were a hospitalization or cytostatic treatment in the 28 days prior to study admission, an ongoing treatment with steroids or immunosuppressant drugs or a freshly diagnosed active form of pulmonary tuberculosis. Pathogen characterization was based on naso-pharyngeal swab, blood culture or sputum sampling. A majority of approximately 60% of all study participants with viral pneumonia were exclusively infected with SARS-CoV-2. Blood collection was performed using a 7.5 mL EDTA blood collection tube. Samples for lipidomic, metabolomics and proteomic analysis were collected at baseline, as well as after a follow-up of three and seven days. Baseline samples were aimed to be taken from patients naïve to antibiotics. In three cases of bacterial infection and eight cases of viral infection an empiric antibiosis had been started before sample collection. The exact follow-up timeline for all samples is included in Figure S1 of the supplementary material. The sampling protocol mandated a centrifugation at 3000 g for 15 min with subsequent storage at 4–8 °C until freezing the samples at -80 °C before and during transfer to the biobank. Centrifugation was to be performed immediately, but no later than 6 h after sample collection. Samples were stored at -80 °C until analysis and shipped on dry ice.

### Lipidome and Metabolome analysis

Sphingoid bases (8), ceramides (22) and eicosanoids (11) were analyzed as previously described in detail^22,23^. Briefly, analytes were extracted from plasma samples by liquid-liquid extraction or solid phase extraction and analyzed using quantitative liquid-chromatography tandem-mass spectrometry assays (LC-MS/MS). Quantification was performed using external calibration in combination with analyte-individual isotopically labelled internal standards resulting in absolute quantitative data.

Furthermore, a standardized set of 630 lipids and polar metabolites was additionally measured using the MxP Quant 500 kit (biocrates life sciences AG, Innsbruck, Austria). Analyses were performed using an LC-MS/MS system consisting of a QTRAP 6500 + triple quadrupole mass spectrometer (Sciex, Darmstadt, Germany) with a Turbo Ion Spray source in electrospray ionization mode, and an Agilent 1290 Infinity LC system featuring a binary HPLC pump, column oven, and autosampler (Agilent, Waldbronn, Germany). Both LC-MS/MS and flow injection analysis MS/MS (FIA-MS/MS) were conducted according to the manufacturer’s protocol, resulting in relative quantitative lipid and polar metabolite data of arbitrary unit. For sample preparation, 10 µl of all samples, including QC samples, calibrators, zero samples, and project samples, were pipetted onto the filter inserts of a 96-well plate. The samples were dried under a gentle stream of nitrogen for 30 min, followed by derivatization with 50 µl of PITC solution at room temperature for 60 min. After derivatization, the samples were dried again for 60 min under nitrogen. To extract the metabolites, 300 µl of a 5 mM ammonium acetate solution in methanol was added to each well, and the plate was shaken for 30 min. The extracts were collected through centrifugation of the plate. Finally, the extracts were diluted for analysis: for FIA, a dilution of 1:50 with running solvent was used, and for LC-MS/MS analysis, a dilution of 1:1 with water was applied.

Afterwards, data were merged by their sample identifiers to obtain the whole lipidomics and metabolomics dataset after data cleaning (see 3.4.2). Six ceramide was determined twice by two of the aforementioned methods and was removed from the results of the MxP Quant 500 data.

### Proteome analysis

Analysis of the plasma proteome was performed using proximity extension assay (PEA) technique using the Olink Target 96 platform (Olink, Uppsala, Sweden). Here, the inflammation, immune response and organ damage panels were utilized to investigate the relative concentration of 275 systemic protein, including the duplicate determination of four proteins. Plasma samples were processed and analyzed according to the manufacturer’s protocol. Signals were processed in Olink NPX manager v3.0.1 (Olink, Uppsala, Sweden). Resulting continuous protein abundances are derived from protein-specific quantitative polymerase chain reaction (qPCR) of arbitrary unit and were reported as log_2_-transformed NPX values.

### Theory and calculations

#### Software

Calculations were conducted in R version 4.3.1 (R Foundation for Statistical Computing, Vienna, Austria) using the development environment RStudio version 2022.02.3 Build 492 (RStudio, Boston, MA, USA). The dependencies included were “dplyr”^24^, “rcompanion”^25^ and “reshape2”^26^ for data wrangling, “ggplot2”^27^, “ggforce”^28^, “EnhancedVolcano”^29^ and “pheatmap”^30^ for visualization and “MatchIt”^31^, “rstatix”^32^, “caret”^33^, as well as ”mixOmics”^34^ for statistics and machine learnings. Lipid network analysis was performed using LINEX2 (date of access: 27th of September 2024)^35^. The Reactome pathway database V86 was used for protein pathway analysis (date of access: 2nd of October 2024 )^36^. MetaboAnalyst V6.0 was used for PLS-DA validation^37^. Adobe Illustrator CS 5.1 (Adobe Inc., 2019) was used for illustrating the output of lipid network enrichments. Adobe InDesign CS5.5 (Adobe Inc., 2019) was used in compositing plots.

#### Data processing

Metabolomics, lipidomic, as well as proteomic analytes were assessed for the rate of data points missing due to falling below the limit of detection (LOD), or the limit of quantification (LLOQ) respectively. All analytes with > 20% of missing values were omitted from the datasets. Missing values in the metabolomics and lipidomics datasets were then imputed with half of the LOD or LLOQ, respectively. For the proteomics OLINK panels the values below the LOD were kept according to the manufacturer’s recommendation. No batch effects were detected. Within the lipidomic and proteomics dataset each analyte was investigated for outliers, which were defined as lying 1.5*inter-quartile ranges (IQR) below the first quartile or 1.5*IQR above the third quartile. Samples that were identified to contain outliers in over 80% of all measured analytes within their respective dataset were omitted from further analysis. Before hypothesis testing, data were power-transformed using Tukey’s ladder of power^38^. The resulting dataset contained 462 lipids and metabolites as well as 146 proteins across 172 samples.

#### Statistical analysis

Analysis was performed with a focus on aspects of the human plasma lipidome, metabolome and proteome differentiating viral from bacterial pulmonary infections. Possible differences between CAP caused by COVID-19 or other viral pathogens were investigated as well. In testing for typical clinical confounders, an unpaired Student’s t-Test was calculated for age differences after propensity score matching. A Chi-Squared test was calculated for assessing differences in gender distribution between the two groups. Data from OLINK-methods were natively reported as dimensionless, log_2_-transformed NPX-values. For differential expression analysis the mean fold change between the two groups were calculated using non-logarithmized data, in order to derive the average change in expression of analytes, between the bacterial and viral cohort in percent. A group- and analyte-wise Shapiro-Wilkes-Test was performed for assessing normality in the transformed data (supporting information, Table S2 and S5). An analyte-wise Levene’s-Test was performed to check for homoscedasticity between the two groups (supporting information, Table S3 and S6). Based on the results of assumption testing a student’s t-Test was performed for differential expression analysis comparing the two group in baseline samples. In the following text, significance is presented at an alpha-Level of α = 0.05. Fold changes of > 20% in either direction are additionally highlighted, exceeding the threshold for commonly allowed variation in quality control samples in bioanalytical methods. Effect size and hypothesis testing were summarized in volcano plots. This method facilitated the determination of the most relevant analytes. Group comparisons of significantly altered analytes were repeated on follow-up sample collection instances and are presented as boxplots. Resulting p-Values are presented as 0.05 > * > 0.01 > ** > 0.001 > *** > 0.0001 > ***. This visualization aimed to assess whether the trends observed at baseline persisted over the following days. To adjust for the error due to multiple testing, resulting p-Values from the t-tests were adjusted using false discovery rate (FDR) (Table S1 and S4).

#### Machine learning

Principal component analysis (PCA) was performed on z-transformed lipidomic, metabolomic and proteomic datasets for assessing, whether the type of pathogen or the severity of infection were among of the main drivers of variation in the respective datasets^39^. For both omics datasets (metabolomics/lipidomics and proteomics) the first and second principal components (PC1/2) were plotted and individual points were colored to reflect infection type and disease severity according to the CRB-65-score^40^.

Partial-least squares discriminant analysis (PLS-DA) was further used for dimensionality reduction and identifying features distinguishing most readily between the bacterial and viral infection^41^. The relative importance of features included in the first latent component was calculated by scaling their coefficients to 100% ad subsequently displayed in form of a bar plot. For model validation the plots were recreated using MetaboAnalyst and autoscaled features^37^.

For integration of lipidomics, metabolomics and proteomics information, all analytes which were identified to be expressed significantly different were z-transformed and clustered using the Euclidean distance norm, combined agglomerative hierarchical Ward’s clustering. The resulting dendrogram is displayed alongside a heatmap encoding the scaled concentrations. A break was inserted indicating the two clusters with lowest respective intra-cluster distance.

#### Proteomic pathway analysis and lipid network enrichment

Proteomic pathway analysis was performed using the “Pathway Analysis with Down-weighting of Overlapping Genes” (PADOG) implemented in the Reactome database^42^, which weighs the relevance of identified proteins relative to how common they appear in gene sets. Interactors were excluded in the analysis. The ten pathways with the highest expression in either of the groups were summarized in a scatter plot with point size indicating the negative decadic logarithm of p-Values associated with their expression.

Lipid network exploration was performed using LINEX2^35^. Analyte nomenclature was converted to LipidLynxX^43^. The full lipid network can be obtained from the supporting information (Figure S2). Lipid network enrichment was run for the identification of relevant lipid subnetworks with properties discriminating between bacterial and viral pulmonary infection. Other settings were kept as default. Resulting subnetworks were illustrated in Adobe Illustrator. The original graph can be obtained from the supporting information (Figure S3).

## Results and discussion

### Study population

To provide a comprehensive understanding of the study findings, it is essential to first examine the demographic and clinical characteristics of the study cohort. The age of study participants ranged from 26 to 90, with a median of 63 years. Overall, 25 female and 44 male subjects were included (Figure S1, Table S1). Two patients died as a result of their infection. The initial study cohort displayed a significant imbalance in age in combination with bacterial infection (p-Value of 0.0007 according to Wilcoxon’s Rank Sum test). This was circumvented by age-matching viral and bacterial cases using a generalized linear model for propensity score matching, as the distinction of viral and bacterial respiratory infections was among the main aims of this study. This resulted in the inclusion of data from 58 out of the initial 69 patients, with no significant differences in age distribution between the groups (p-Value = 0.2 according to Wilcoxon’s Rank Sum test, Table 1). Among these patients, 18 suffered from bacterial CAP, while the other 40 were infected with viral pathogens. The viral cohort consisted of 22 cases of SARS-CoV-2 and 18 cases of other viral pathogens. Microbial investigation led to the identification of *Streptococcus pneumoniae*, *Staphylococcus aureus*, *Haemophilus influenzae*, *Moraxella catarrhalis*, *Legionella pneumophila*, *Legionella longbeachae*, and *Mycoplasma pneumoniae* as pathogens among the bacterial cohort. Viral CAPs were caused by SARS-CoV-2, *Influenza A* (specifically H1N1 in certain cases) *& B*, *Respiratory Syncytial Virus A & B*, *Human Metapneumovirus A & B*, *Human Coronavirus* (HKU1 and NL63), and *Rhinoviruses* (please refer to Figure S4 for a closer inspection of pathogen distribution). There was no significant difference in gender distribution between the viral and bacterial infection groups (p-Value = 1, Table 1).

**Table 1:** Descriptive statistics on investigated CAP cohort. Self-reported gender is given in male (M) and female (F).

	Viral		Bacterial	
N	40		18	
Age [years]	55.2 ± 16.0		61.2 ± 18.0	
Gender	Male	26	Male	12
	Female	14	Female	6
Duration of hospital stay [days]	11.2 ± 8.1		13.4 ± 8.2	
ICU admission	Yes	5	Yes	4
	No	35	No	14
CAP severity	Moderate	32	Moderate	7
	Severe	8	Severe	11

Age and the duration of the hospital stay are given in mean and standard deviation. ICU admission was counted, if a patient had to be treated at the ICU within at least one of the three explored visits. Moderate cases were defined as patients who were hospitalized but did not require a stay in the ICU or IMC. Severe cases were defined as patients who were hospitalized and required a stay in the ICU or IMC. The 58 patients were selected from the initial 69 patients through propensity score matching, ensuring that no statistically significant differences in age and gender were observed with regard to the types of infections.

To investigate day-to-day variabilities of the molecules the trends identified through omics analyses, follow-up samples were obtained from the same patients three and seven days after study admission. However, it has to be noted that the disease course and inter-individual biological variance can influence this longitudinal comparison. A follow-up sample for each patient was obtained on the third day after study admission, with the number of samples decreasing to roughly 50% at the seven-day follow-up. This reduction in sample size was mostly due to missing samples from individuals with moderate viral infection, who were free to leave in-house treatment within the first week after admission.

### Lipidomic and metabolomic analysis

**Fig. 1:**
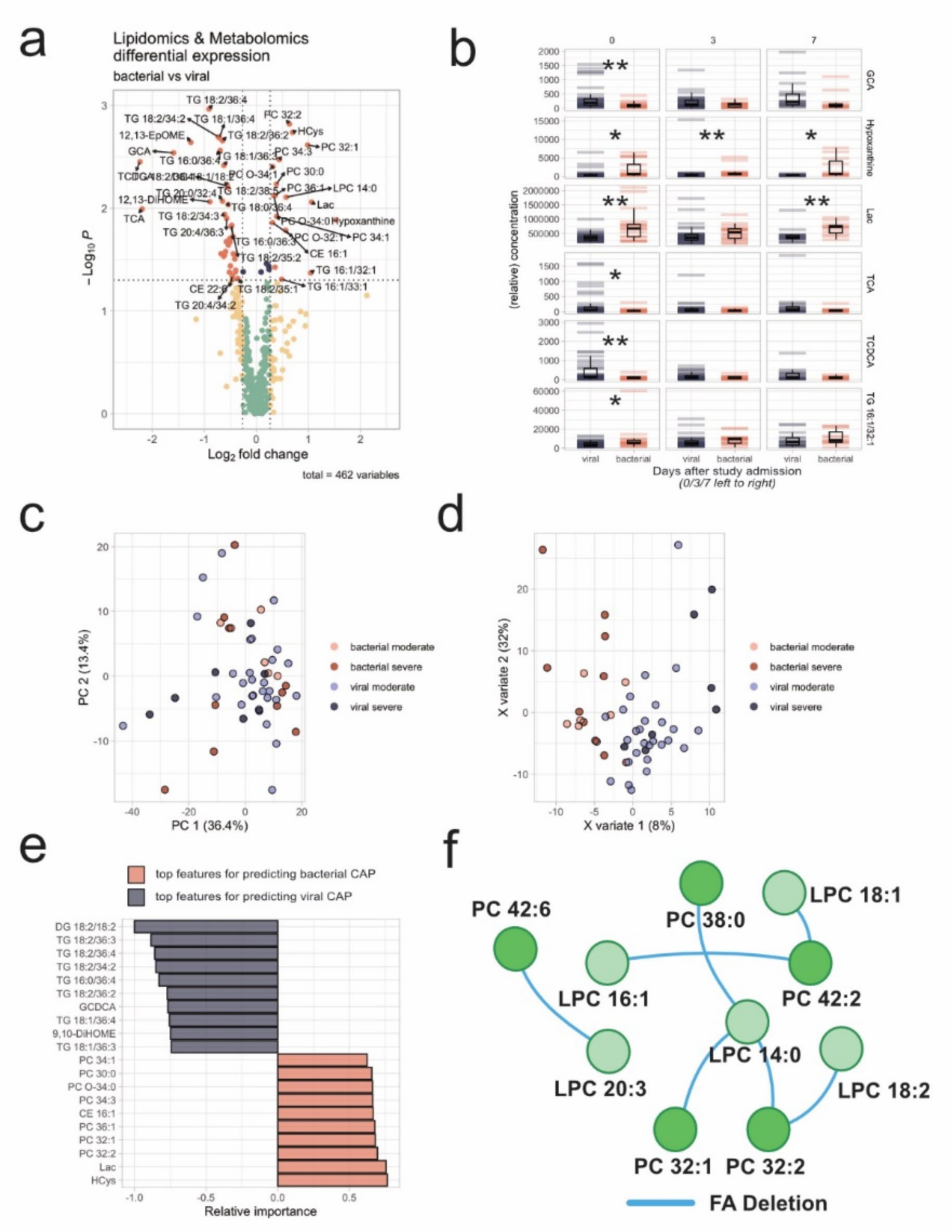
Metabolomic and lipidomic analyses – 1 (**a**): Volcano plot with differentially abundant lipids and metabolites. Vertical cut-offs indicate a p-Value below 0.05. Horizontal cut-offs indicate a mean fold change of 20% towards bacterial infection (right) or viral infection (left); 1 (**b**): Group comparison of selected lipids and metabolites with results of unpaired t-Tests across sampling instances (Day after admission on top. The exact sampling time points in relation to the baseline of patient admission can be obtained from Figure S1). Lipids are presented in an arbitrary unit; 1 (**c**): Scatter plot of the first two PC of the whole lipidomics/metabolomics dataset, color-coded for pathogen type and severity; 1 (**d**): Scatter plot of the first two latent components of PLS-DA, color-coded for pathogen type and severity; 1 (**e**): Ten most important features each differentiating between viral and bacterial infection within the first latent component of the PLS-DA, scaled to 100%.; 1 (**f**): Lipid subnetwork identified by lipid network enrichment.

Throughout the analysis, lipidomics and metabolomics data were analyzed separately from the proteomics data. The combined lipidomics and metabolomics data were visualized using a volcano plot (Fig. 1a) to identify the most relevant analytes differentiating bacterial and viral CAP at baseline. Boxplots for these analytes were generated to assess whether the observed trends persisted over subsequent visits (Fig. 1b). A PCA was performed as an unsupervised method but did not clearly separate the groups (Fig. 1c), leading to the use of PLS-DA for better group differentiation and identification of key analytes (Fig. 1d, e). Validation of the PLS-DA model with MetaboAnalyst indicates potential overfitting (Figure S6). Therefore, variable importance was only used for feature reduction. This approach aims to identify potentially relevant analytes, which can then be discussed in the context of already published studies. Finally, the lipidomics-focused tool LINEX was used to identify significant changes in the lipid network associated with different forms of CAP (Fig. 1f).

Within the lipidomic profile at baseline, several lipids displayed notable differences between viral and bacterial pneumonia (Fig. 1a, b) including the bile acids glycocholic acid (GCA), taurocholic acid (TCA) and taurochenodeoxycholic acid (TCDCA) as well as various tri- and diglycerides (TGs and DGs), and several phosphatidylcholine (PCs). The analysis of the dataset using PLS-DA (Fig. 1d, f) highlights a similar trend compared to the volcano plots (Fig. 1a). Indeed, some differences in the data could be observed between groups of COVID-19 patients and other viral CAP (Figure S5). However, a bigger patient cohort will be necessary to validate and further investigate those differences.

**Fig. 2:**
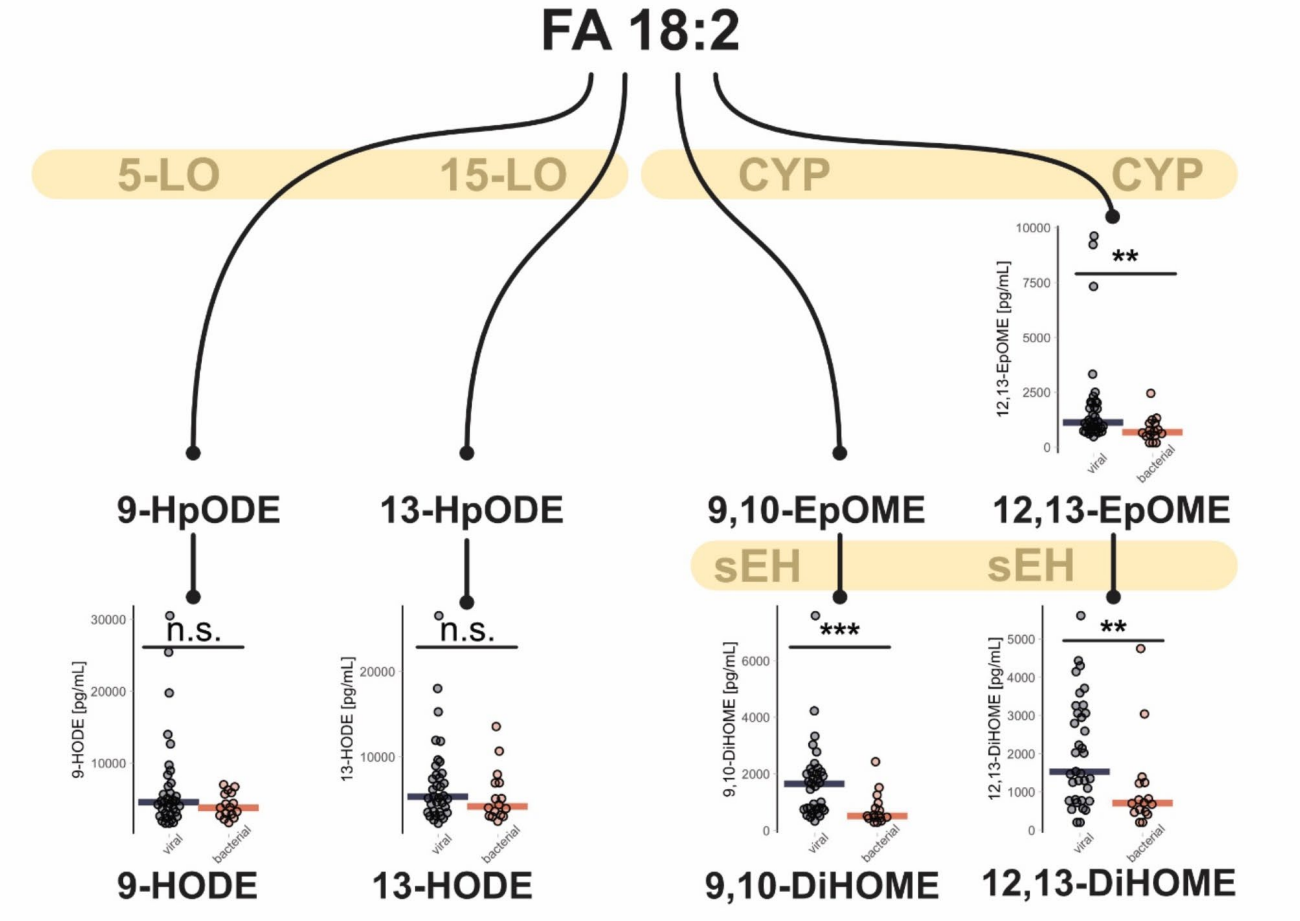
Oxylipin formation from linolenic acid – Scatterplot representing individual sample concentrations, with a horizontal line representing the median. While lipoxygenases-derived linolenic acid metabolites displayed moderately increased effect sizes in the viral infection group, larger and significant changes were observed in the CYP2A2-derived 12,13-EpOME, 12,13-DiHOME and 9,10-DiHOME. Here, Student’s t-Tests with p-Values unadjusted for multiple testing compare the difference in means of viral and bacterial pneumonia. Notably, 9,10-DiHOME remains significant after FDR adjustment, as shown in Table S1.

In bacterial CAP, patients primarily showed increased levels of various PCs and ether-PCs (PC-O), which was confirmed by lipid network enrichment, leading to the identification of a subnetwork (Fig. 1f) focusing on fatty acid transfer between PCs and lyso-PCs (LPCs). In contrast, in viral CAP, various TGs and DGs are primarily elevated, with the notable observation that these are mainly TGs and DGs containing the fatty acid 18:2 (FA 18:2) at least once. This observation is particularly interesting due to the differences in the levels of the FA 18:2 metabolites 12,13-Epoxy-octadecenoic acid (12,13-EpOME) and 9,10-Dihydroxy-octadecenoic acid (9,10-DiHOME) between viral and bacterial CAP. Consequently, these oxylipins have been re-examined in detail. In contrast to EpOMEs and DiHOMEs, other FA 18:2-derived signaling oxylipins of the Hydroxyoctadecadienoic acid (HODE) type were not altered significantly between the viral and bacterial infections groups (Fig. 2). This suggests that the increase of CYP450-derived oxylipins in the viral cohort is not due to differences in intake of FA 18:2, which is the most abundant polyunsaturated fatty acid in western diet^44^. Prior work has associated viral infections with lowered concentrations of FA 18:2^45^. The metabolism to its inflammation signaling derivates offers an explanation to these findings. In-vitro experiments were further able to demonstrate an increase in EpOME and DiHOME concentrations in virally stimulated cells^46^. The present data further expand these findings in comparison to bacterial CAP. The 12,13-DiHOME and 12,13-EpOME results revealed an excess of the dihydroxylated oxylipin compared to its epoxide form, indicating high soluble epoxide hydrolase (sEH) activity. Inhibition of sEH has been discussed to be a viable mode of combating pulmonary inflammation caused by tobacco smoke exposure^47^. DiHOMEs have previously been associated with lung damage as a consequence of influenza infections, causing edema formation and decreased alveolar perfusion^44^. Besides displaying anti-inflammatory properties elsewhere^48^, the sEH products’ mechanism in causing lung damage might be explained by impairing mitochondrial function^44,49^.

Since pre-analytics are particularly important for lipidomics analyses, the stability of the identified analytes was tested. Stability testing has proven altered lipids (Fig. 1a), especially the relevant DiHOMEs and 12,13-EpOME, to be stable in whole blood samples at room temperature for at least four hours^22,50^. For TG, PC, and PC-O, even longer storage durations were proven to be stable.

### Proteomic analysis

**Fig. 3:**
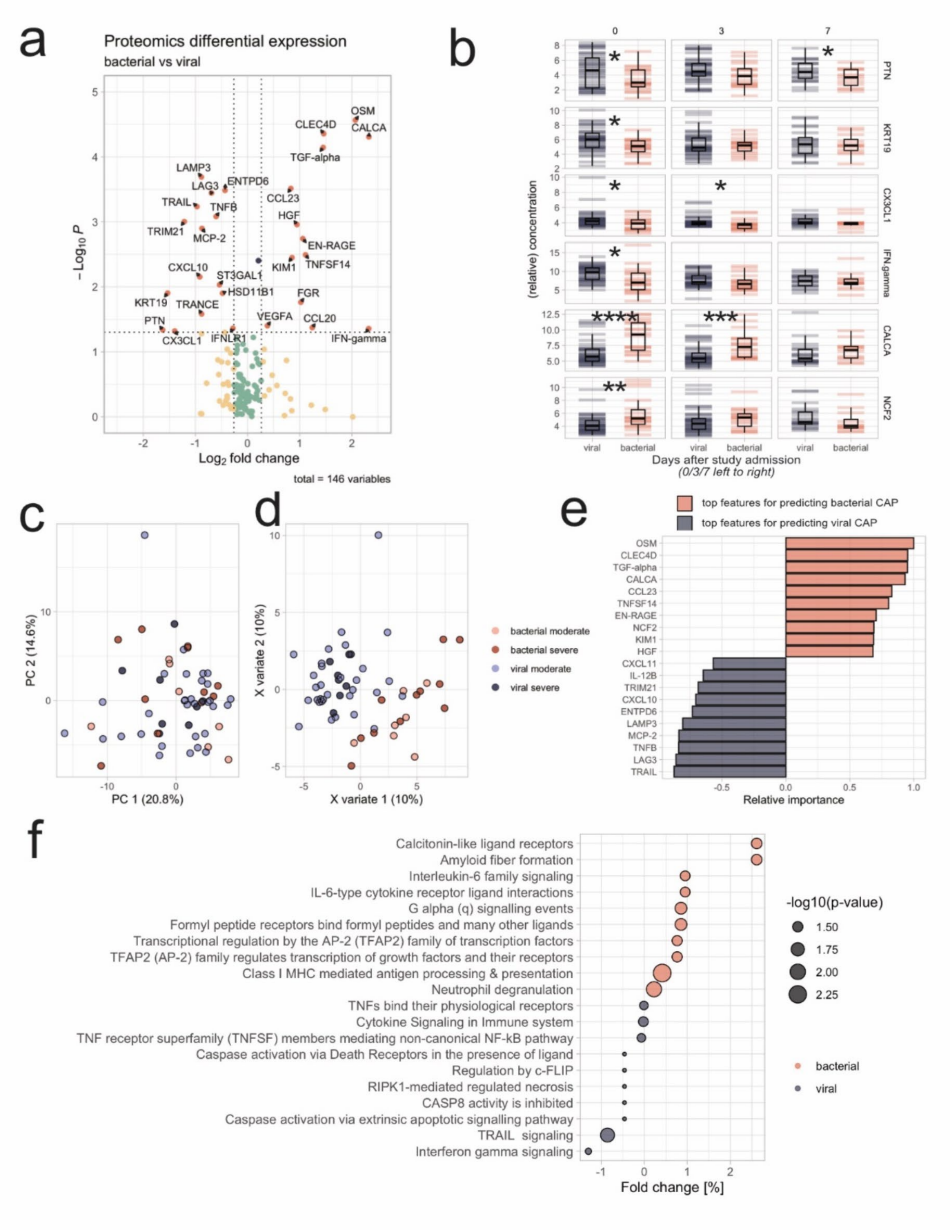
Proteomic analyses – 3 (**a**): Volcano plot with differentially abundant proteins. Vertical cut-offs indicate a p-Value below 0.05. Horizontal cut-offs indicate a mean fold change of 20% towards bacterial infection (right) or viral infection (left); 3 (**b**): Group comparison of selected proteins with results of unpaired t-Tests across sampling instances (Day after admission on top. The exact sampling time points in relation to the baseline of patient admission can be obtained from Figure S1). Proteins are presented in arbitrary units; 3 (**c**): Scatter plot of the first two PC of the whole proteomics dataset, color-coded for pathogen type and severity; 3 (**d**): Scatter plot of the first two latent components of PLS-DA, color-coded for pathogen type and severity; 3 (**e**): Ten most important features each differentiating between viral and bacterial infection within the first latent component of the PLS-DA, scaled to 100%.; 3 (**f**): Top 10 over-expressed pathways for each group resulting from PADOG gene set analysis.

Proteomics data was analyzed independently from the lipidomics and metabolomics data following similar steps as described before. These included the visualization of data with volcano plots (Fig. 3a), the generation of boxplots for relevant analytes (Fig. 3b), and the use of PCA and PLS-DA for group separation (Fig. 3c, d, e). Instead of using LINEX, the final step for the proteomics data involved identifying the top 10 over-expressed pathways for each group resulting from PADOG gene set analysis (Fig. 3f). The analysis of the proteome yielded several proteins displaying significant differences between viral and bacterial pneumonias (Fig. 3a). A comprehensive list of fold changes, including all results of hypothesis testing, is partially presented in Fig. 3b and fully detailed in the supplementary material. Just like observed in the lipidomic and metabolomics dataset, PCA was able to show that the distinction between viral and bacterial pathogens did not predominantly account for the variance of the proteomic data (Fig. 3c). Variable importance of a discriminatory PLS-DA (Fig. 3d, e) confirmed most of the proteins from the volcano plot (Fig. 3a) being relevant drivers in distinguishing the two different cohorts. Yet, it has to be noted that overfitting of the PLS-DA model is likely (Figure S7). Reactome pathway enrichment identified various pathways affected differentially between viral and bacterial pneumonia (Fig. 3f).

A comparison of the data and identified proteins obtained in this study with previously published data shows that some proteins have already been identified as relevant in other studies, which increases the reliability of the results from this study. For example, tumor necrosis factor-related apoptosis-inducing ligand (TRAIL) has been identified as a part of a biomarker panel to distinguish between viral and bacterial infections in children^51^. In another study, hepatocyte growth factor (HGF) and TRAIL were identified as relevant proteins for distinguishing community-acquired pneumonia (CAP) from healthy controls^52^. Interestingly, our data further specifies TRAIL as a predominantly viral CAP-associated protein while HGF correlates with bacterial CAP. Previous studies could show that elevated levels of oncostatin M (OSM) can serve as a prognostic biomarker for CAP, as the concentration of OSM is closely associated with disease severity and prognosis^53^, which could be corroborated in pneumonia patients^54^. In our study, OSM is one of the top proteins differentiating between viral and bacterial CAP. EN-RAGE, another protein that we can show to be differentially expressed between the two different CAP etiologies, has also been identified as a potential biomarker for predicting the severity and progression of CAP^55^. Due to our findings, this might specifically be true for bacteria-triggered pneumonia. Additionally, another study that was not conducted in plasma or serum found that IFN-gamma, CXCL10, and MCP-2 were significantly elevated in the nasal mucosa of children with viral or bacterial CAP, and CCL23 was significantly elevated in children with viral CAP^56^. Our data suggest that none of those markers, however, has the potential to distinguish between bacterial and viral infections.

### Combination of lipidomic, metabolomics and proteomic data

**Fig. 4:**
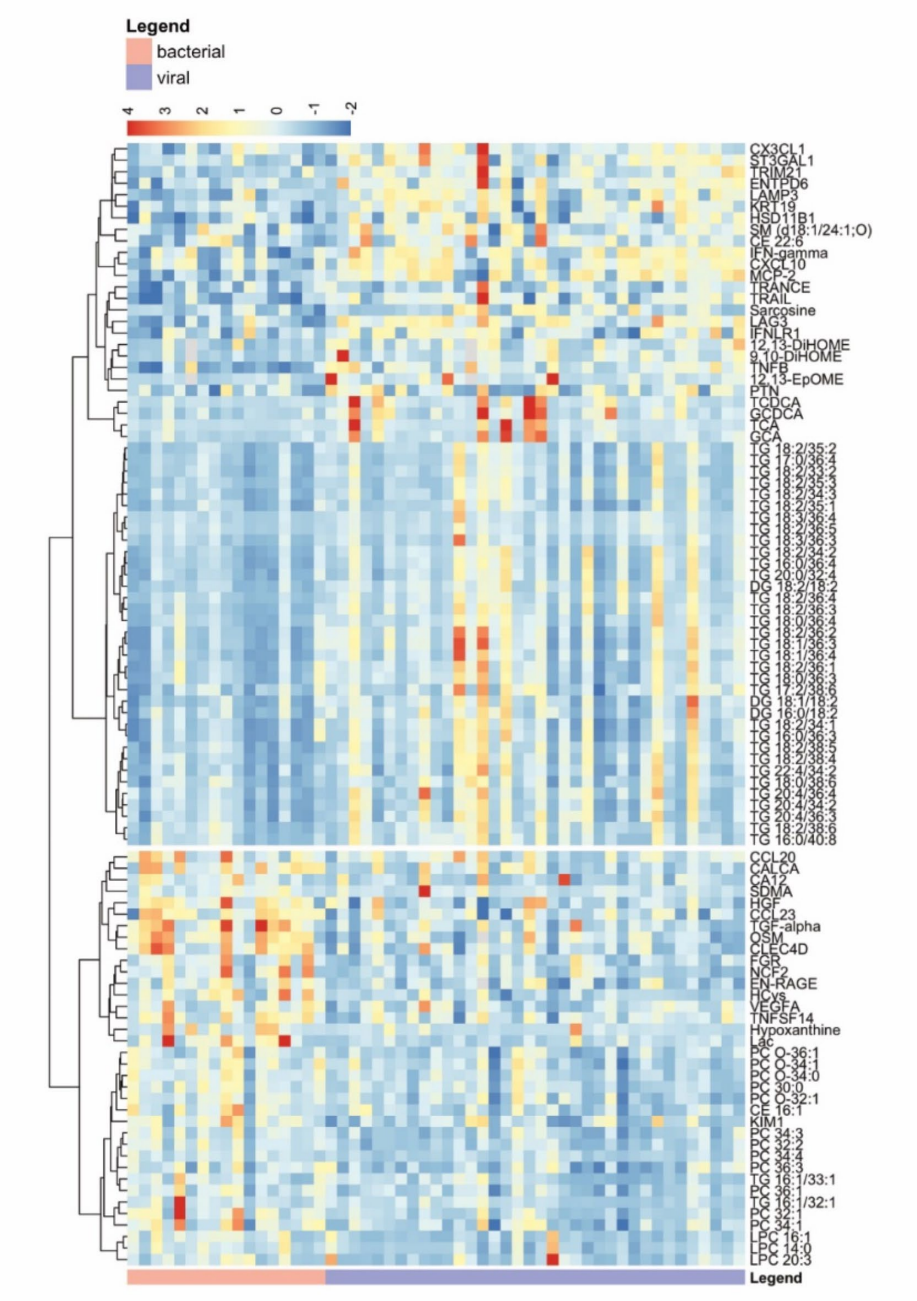
Co-expression of significantly altered lipids, metabolites and proteins – Hierarchical agglomerative clustering of significantly altered lipids, metabolites and proteins. Rows of the heatmap are ordered according to pathogen type. The dendrogram was cut to reveal the two sub-clusters with least within-cluster distance.

Clustering for pairwise distance between lipids, metabolites and proteins with higher abundance in either the viral or the bacterial cohort revealed the co-expression of certain lipids, metabolites and proteins (Fig. 4). Possible relations of certain lipids, metabolites and proteins were identified by assessing their co-expression in either viral or bacterial CAP. We primarily focused on analytes that remained significant after FDR correction (see Supplementary). For bacterial infections the over-expression of CLEC4D of the Dectin-2 family coincided with EN-RAGE receptor (Figs. 3a and 4). Both PRRs are associated with identifying pathogen associated molecular patterns (PAMP) and have previously been revealed as relevant for the immune response in respiratory infections^48,57^. While CLEC4D is specifically triggered by glycolipids, EN-RAGE recognizes a broader range of ligands, such as DNA, RNA, lipopolysaccharides or general lipids^58–60^. Evidence for the role of RAGE proteins in damaging, as well as protecting pulmonary tissue has been found^57^. EN-RAGE has additionally been proposed to be an inadvertent drug target of atorvastatin, explaining parts of its pleiotropic inflammation-reducing properties^61^. The present data contextualizes these findings within CAP, identifying a higher relative abundance of both proteins in bacterial pulmonary infections. Elevated levels of EpOME and DiHOME levels on the other hand concur with increased concentrations of other immune-modulatory proteins, such as TRAIL, LAG-3 or LAMP3 in viral respiratory infections (Fig. 4). Besides being found in plasma, TRAIL has previously been identified as overly abundant in virus-infected cells during influenza infections, where it can induce apoptosis through internal caspase signaling^62^. TRAIL-associated apoptosis has also been identified as one of the pathways overexpressed in the viral infection cohort in comparison to the bacterial infection cohort (Fig. 3f). LAG-3 bears responsibility in T cell modulation after binding to MHC II^63^, a major mechanism of (viral) antigen presentation^64^. Its dysregulation in viral infections has been proposed to be in part responsible for inapt immune response, as well as tissue damage by inflammation^63^. LAG-3 targeted therapy is currently investigated as an adjuvant for vaccines against several virus infections^65^. LAMP3 has also been previously demonstrated to be increased in epithelial lung tissue during infections with influenza A and in SARS-CoV-infections, where the protein promotes autophagy of infected cells^66^. As multiple viral pathogens are responsible for viral CAP in the present study, the data not only confirm these findings but also potentially extend them to other viral infections and delineate differences compared to bacterial infections. As FA 18:2 is a major activator of LAMP3 the present data could further offer insight into a possible mechanism through which the free fatty acid, or more specifically its CYP-/sEH-derivates, could potentially unfold their lung toxicity, by activating autophagy in epithelial cells during viral infection^49^.

While hitherto published studies often focus on specific pathogens, the present multi-centric investigation evaluates lipidomic, metabolomics and proteomic differences between groups of viral and bacterial CAP each consisting of non-homogenous sets of pathogens. Pathogenic diversity within the viral and bacterial cohort are expected to introduce additional variance, thereby strengthening the relevance of analytes that were found to be robust predictors of either of the two infection types. Careful consideration was applied to the selection of included samples, ruling out the possibility of age-related bias in the comparative analysis. The initial analysis of differential expressions of metabolites, lipids, and proteins was conducted on samples taken upon admission to the study and, where possible, trends were verified at subsequent visits. Causal relations between the identified analytes, however, need to be investigated in future work on an external validation cohort, which was unfortunately not possible within the scope of this study and must be considered a limitation. Additionally, there are other limitations that should be acknowledged. Firstly, there is a potential for overfitting, which is why the model was used only for feature selection, and the relatively low statistical power due to the small patient sample size with a slight gender imbalance. Secondly, the absence of a control cohort is a limitation, as it was not feasible to generate such a cohort within this study, and the use of samples from other studies was not possible due to differing pre-analytical protocols. To properly contextualize the findings of the present study, including a healthy control cohort in future work would be beneficial by providing a baseline of analyte concentrations in healthy volunteers.

## Conclusions

CAP poses a significant health threat, particularly among the elderly and immunosuppressed. The urgency for an improved understanding of the inflammatory pathways involved has been highlighted by recent global health challenges. A better comprehension of the distinct systemic inflammatory pathways active during bacterial and viral infections could greatly enhance diagnosis, prognosis, and open avenues for developing effective and specific treatments.

The systemic proteome, metabolome, and lipidome offer promising insights into entities and pathways that differ between viral and bacterial respiratory infections. The present data suggest that linoleic acid-derived oxylipins, specifically of the EpOME and DiHOME type, are active in various viral respiratory infections. Additionally, other lipids such as triglycerides (TGs) and phosphatidylcholines (PCs), as well as various proteins, exhibit differences between viral and bacterial CAP. Several analytes showed overlaps with results from other CAP studies, reinforcing the validity of the findings.

Due to the small sample size with a slight gender imbalance and the absence of a validation cohort within this study, the aim was not to identify definitive biomarkers but to contextualize the data with existing research. This approach enhances the understanding of inflammatory mechanisms that distinguish viral from bacterial CAP and underscores the potential of lipidomic, metabolomic, and proteomic profiling for early differentiation of the two infection types.

Taken together, the present study indicates that several pneumonia-associated markers might be used to distinguish between bacterial and viral CAP. However, further studies analyzing larger sample cohorts will be crucial to confirm the findings and identify the most relevant and stable markers to support patient identification and treatment decisions.

## Electronic supplementary material

Below is the link to the electronic supplementary material.

Supplementary Material 1

Supplementary Material 2

## Data Availability

The datasets generated and/or analyzed during the current study are not publicly available due to reasons for data protection but are available from the corresponding author on reasonable request.
