# Supplementary material for: Proteomic, metabolomic and lipidomic profiles in community acquired pneumonia for differentiating viral and bacterial infections
Source: Sci Rep. 2025 Jan 14;15:1922. doi: 10.1038/s41598-025-85229-2 (PMC11733231; doi:10.1038/s41598-025-85229-2)
Supplement: Supplementary file 2 — Supplementary Material 2 [file 41598_2025_85229_MOESM2_ESM.docx]

Abbreviations

**CAP** – Community-acquired pneumonia, **CCL23** - C-C motif chemokine 23, **CLEC4D** - C-Type lectin receptor 4D, **DG** – Diglyceride, **DiHOME** – Dihydroxyoctadecenoic acid, **EN-RAGE** - Extra-cellular newly-identified receptor for advanced glycation end products, **ENTPD6** - Ectonucleoside triphosphate diphosphohydrolase 6, **EpOME** – Epoxyoctadecenoic acid, **FA** – Fatty Acid, **FDA-NIH** – Federal Drug Administration – National Institute of Health, **FDR** – False Discovery Rate, **FIA** – Flow injection analaysis, **GCA** – glycocholic acid, **HIV** – Human Immunodeficiency Virus, **HODE** – Hydroxyoctadecadienoic acid, **ICU** – Intensive Care Unit, **IMC** – Intermediate Care Unit, **IQR** – Inter-quartile range, **LOD** – Limit of detection, **LAG-3** – Lymphocyte activation gene 3**, LAMP3** – lysosomal-associated membrane glycoprotein 3, **LC** – Liquid chromatography, **LLOQ** – Limit of quantification, **LPC** – Lyso-Phosphatidylcholine, **MS/MS** – Tandem mass spectrometry, **PADOG** - Pathway Analysis with Down-weighting of Overlapping Genes , **PAMP –** Pathogen-associated molecular pattern, **PC** – Phosphatidylcholine, **PC-O** – Ether-Phosphatidylcholine, **PC1/2** – Principal component 1 or 2, **PCA** – Principal component analysis, **PEA** – Proximity extension assay, **PLS-DA** – Partial least squares discriminant analysis, **PRR** – Pattern-recognizing receptor, **qPCR** – quantitative polymerase chain reaction, **SARS-CoV-2** – Severe acute respiratory coronavirus SARS-CoV-2, **sEH** – soluble epoxide hydrolase, **TCA** – Taurocholic acid, **TCDCA** – Taurochenodeoxycholic acid, **TG** – Triglyceride, **TGF-alpha** – Protransforming growth factor alpha, **TNFB** – Lymphotoxin-alpha, **TNFSF14** – tumor necrosis factor receptor superfamiliy member 14, **TRAIL** – TNF-related apoptosis inducing ligand

**
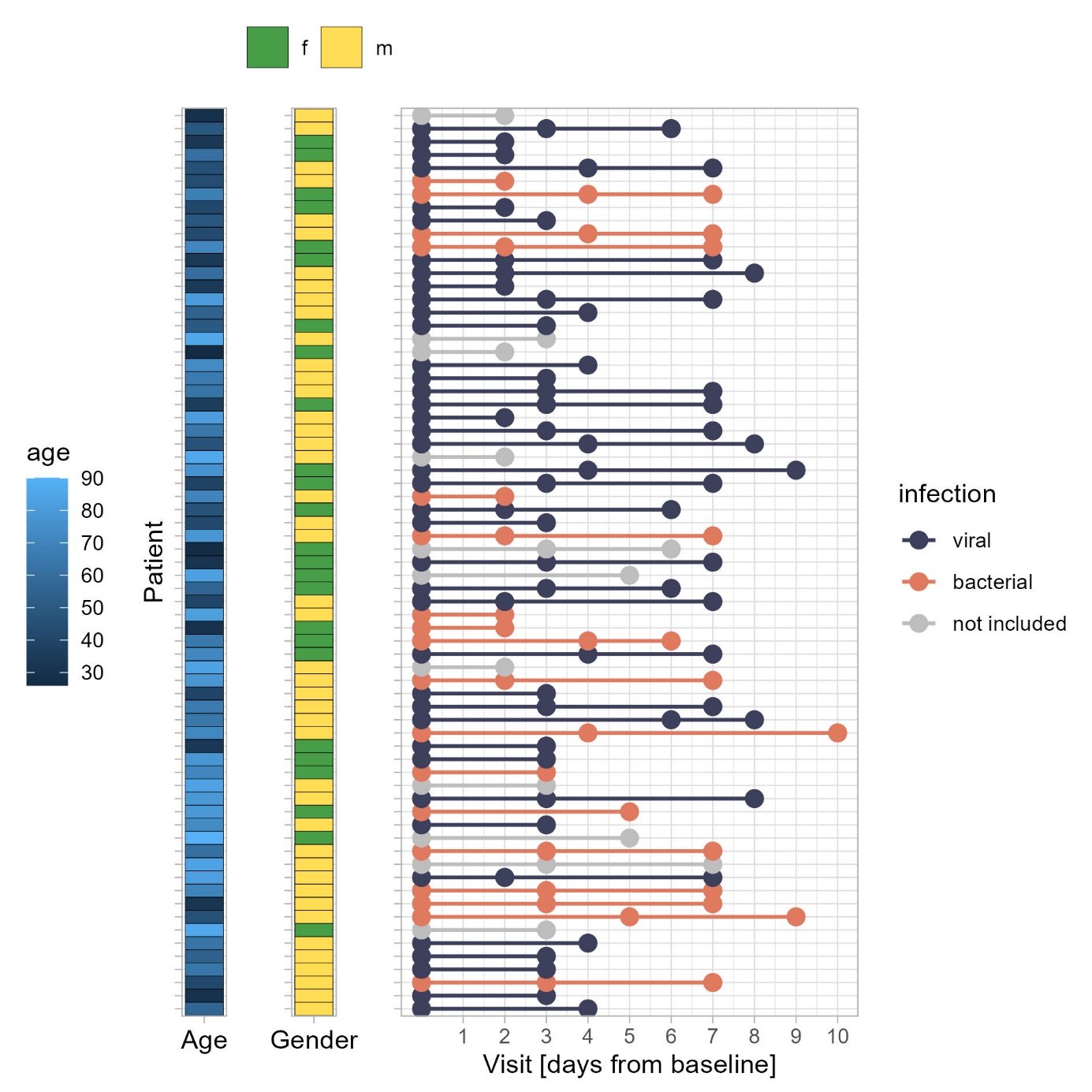
**

Figure S 1 Sample metadata – Patient age varied between 26 and 90 years (blue tile plot). The gender distribution (yellow and green tile plot) within the study was approximately balanced. Up to three samples were analyzed per patient. The first follow up sample was planned on the third day after hospital admission (day 0) but was taken on the second or fourth day in some cases. Approximately fifty percent of second follow up samples, intended to be collected one week after admission, were obtained.


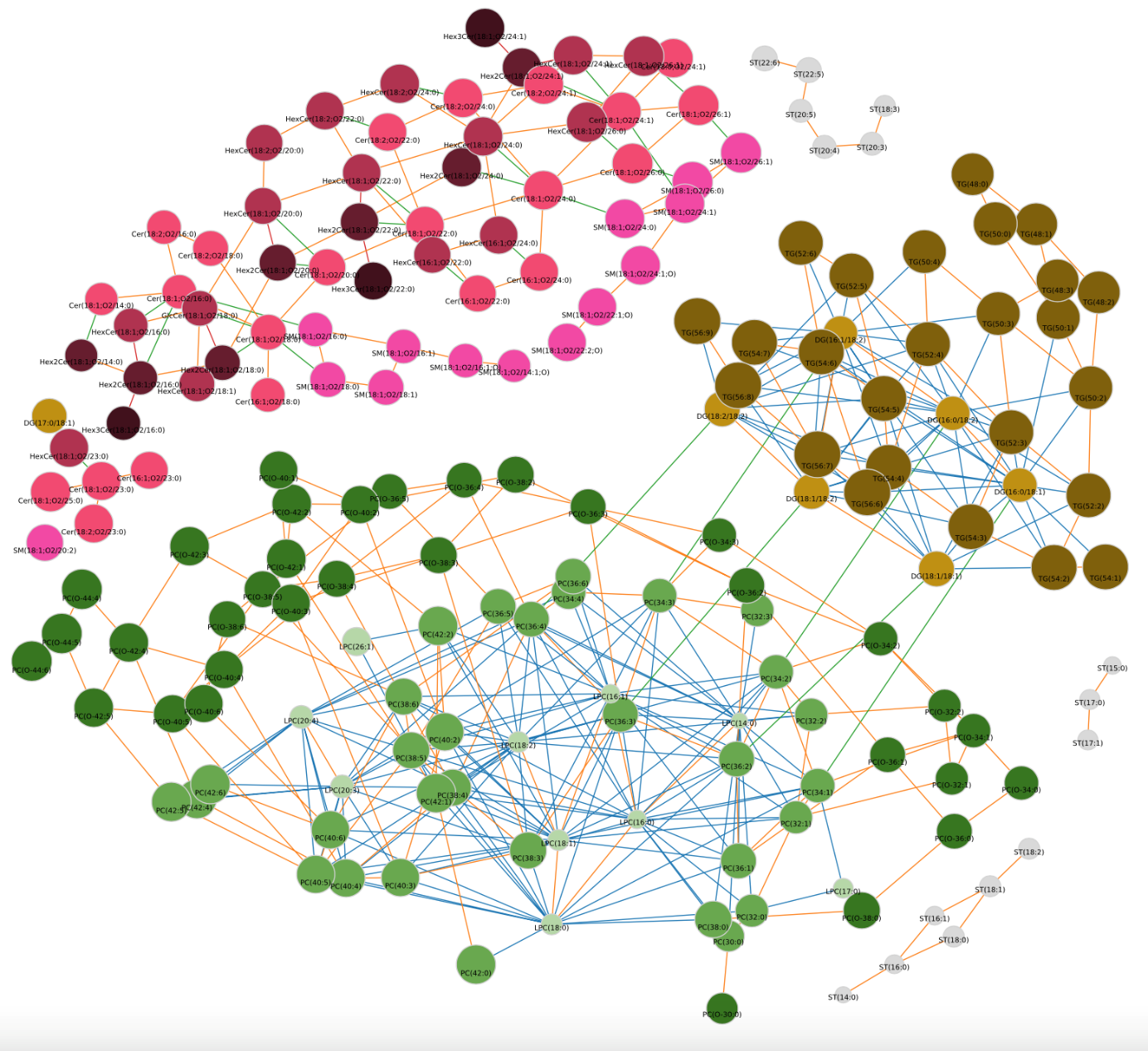


Figure S 2 Whole network view of lipids analyzed with LINEX2. Node size corresponds to carbon chain length. Node color is assigned based on lipid (sub) class. Color of edges gives information on reactions between lipid species.


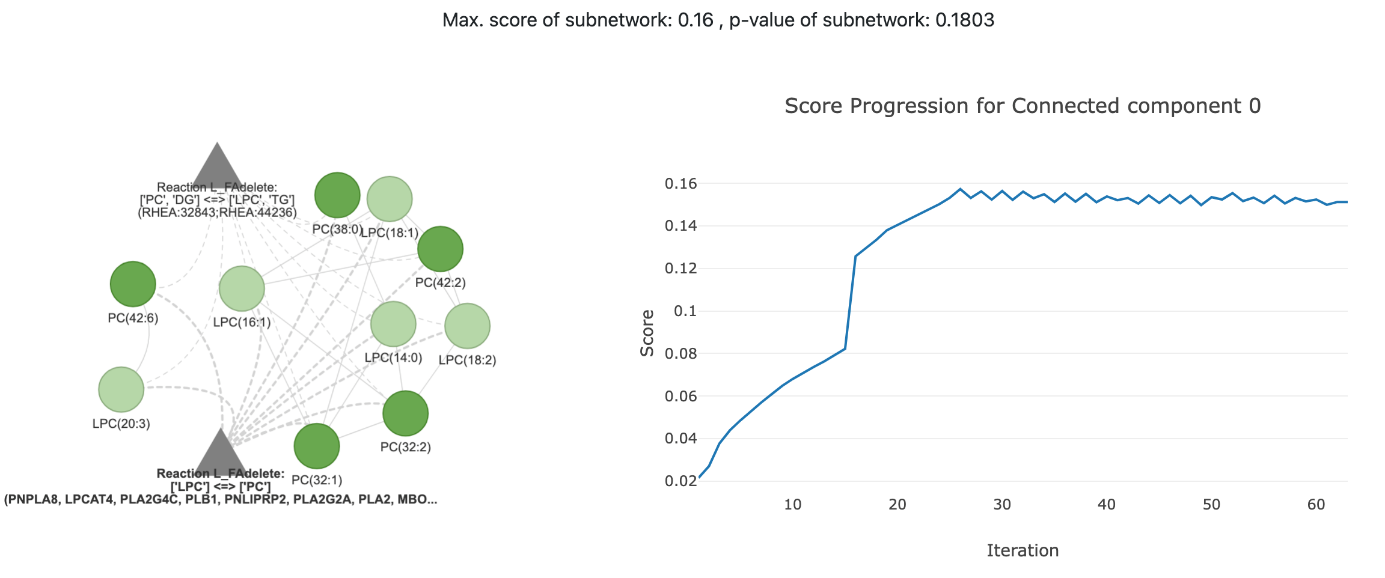


Figure S 3 First local lipid subnetwork identified by the LINEX2 lipid network enrichment algorithm (first panel). The iterative score optimization is displayed in the second panel. Depiction of the subnetwork in the main manuscript are based on the first panel.


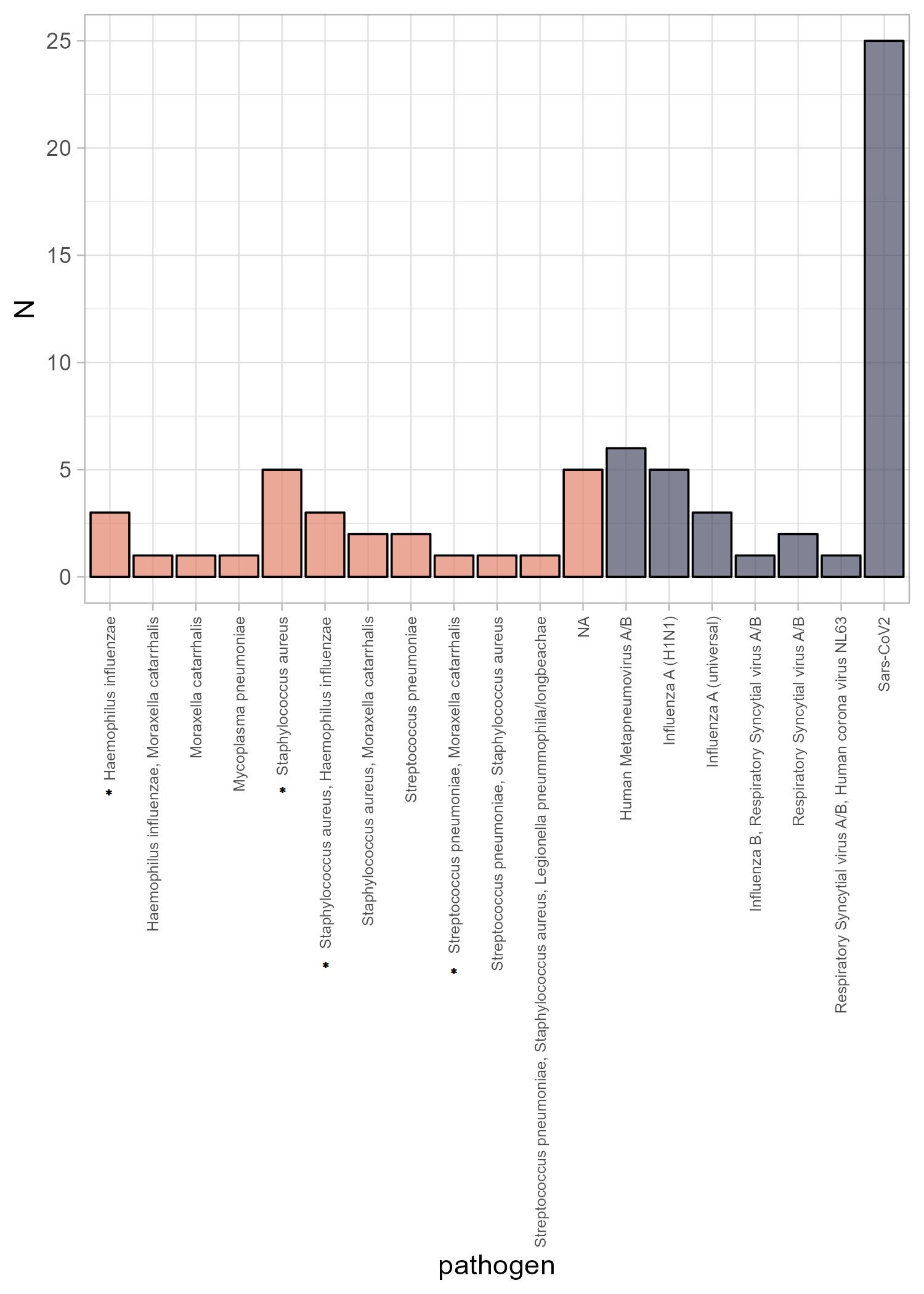


Figure S 4 Pathogen distribution among groups of viral and bacterial CAP within the present study. If multiple pathogens were detected within a patient they are presented as the respective combination. *Positive tests for either Rhinovirus or the Human Coronavirus HKU1 were additionally detected in four patients with bacterial CAP, but where deemed incidental and not responsible for the respective CAP.


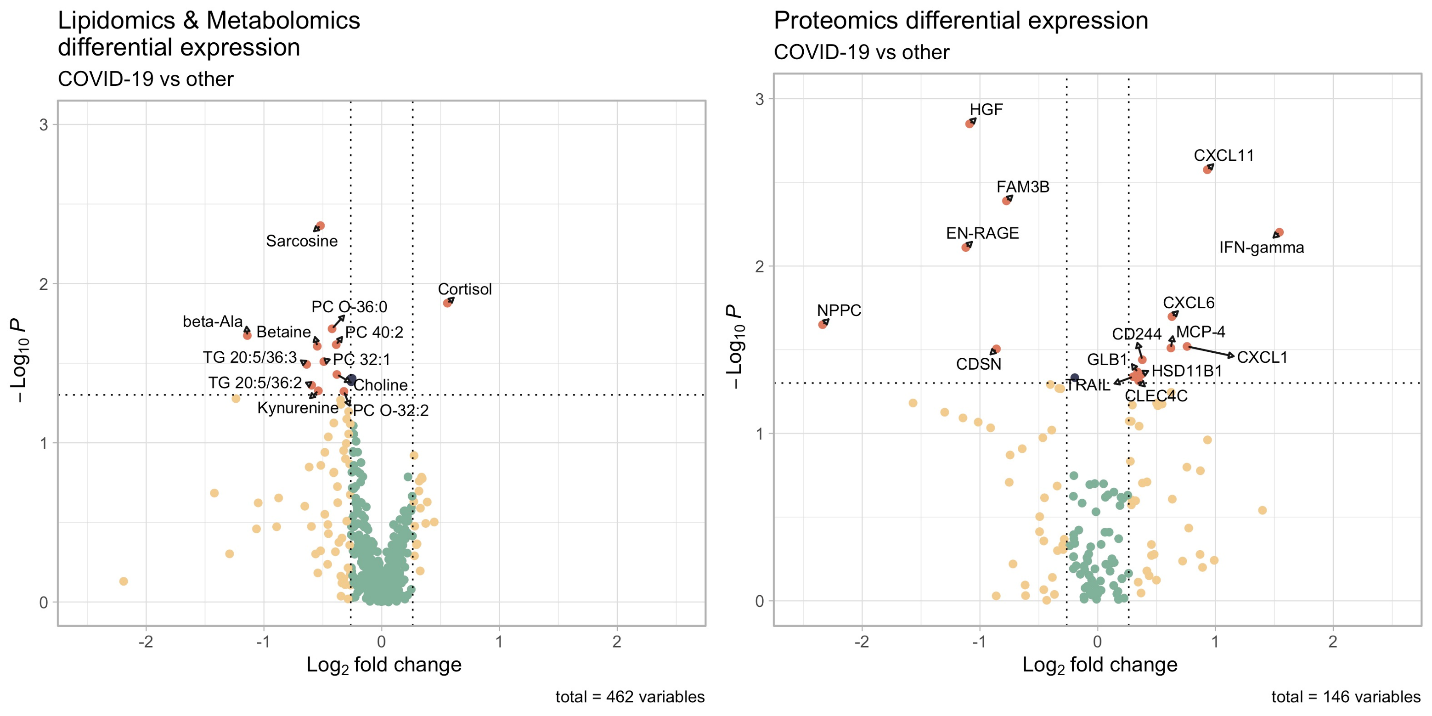


Figure S 5 Differential expression analysis of the systemic lipidome comparing viral community-acquired pneumoniae with and without Sars-CoV2 infections. Fold change thresholds are displayed as the log_2_ of 20 % in- or decrease in concentration. The threshold of the alpha-Level was set to the –log_10_ of α = 0.05.


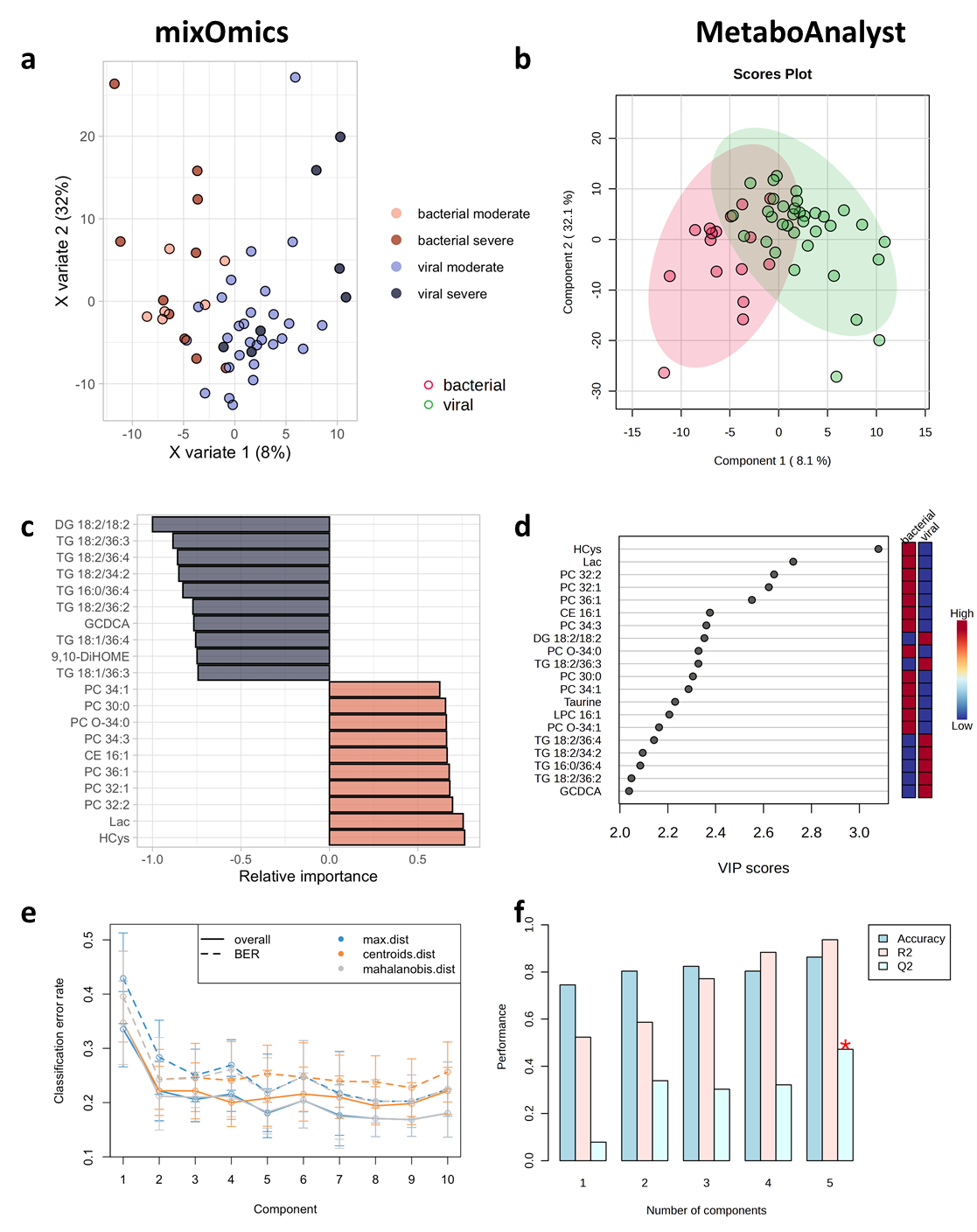


Figure S 6 Lipidomics PLS-DA model comparison between mixOmics and MetaboAnalyst. For validation purposes the PLS-DA model created with the R library mixOmics was recalculated in MetaboAnalyst v6.0, offering a different set of validation metrics. Slight differences are present but were expected. a+b: 2D-Scores Plot from mixOmics (a) and MetaboAnalyst (b), please note the mirrored orientation. c: Ten most important features each differentiating between viral and bacterial infection within the first latent component of the mixOmics PLS-DA model, scaled to 100 %. d: 20 most important features for the differentiation between bacterial and viral infection, absolute variable importance scores. Increase or decrease in the respective group is displayed on the right. e: M-fold cross validation of the mixOmics model (folds = 3). Differences between the overall classification error rate and the balanced error rate (BER) show a disbalance in the group sizes. f: Leave-one-out cross validation in MetaboAnalyst, good predictions will have a high R2 and Q2; a lower Q2 or a gap between R2 and Q2 of more than 0.3, means that the model is potentially overfitted.


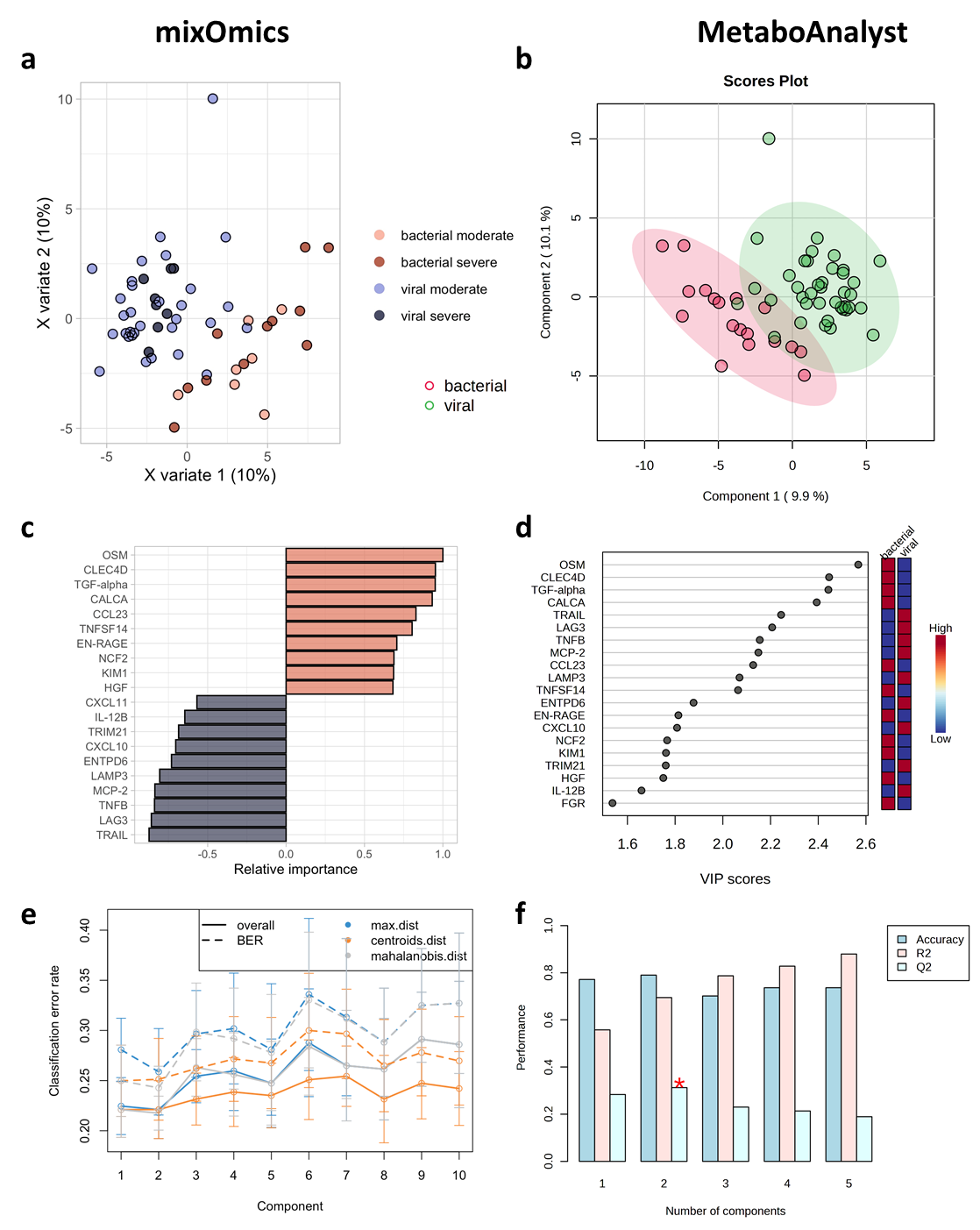


Figure S 7 Proteomics PLS-DA model comparison between mixOmics and MetaboAnalyst. For validation purposes the PLS-DA model created with the R library mixOmics was recalculated in MetaboAnalyst v6.0, offering a different set of validation metrics. Slight differences are present but were expected. a+b: 2D-Scores Plot from mixOmics (a) and MetaboAnalyst (b), please note the mirrored orientation. c: Ten most important features each differentiating between viral and bacterial infection within the first latent component of the mixOmics PLS-DA model, scaled to 100 %. d: 20 most important features for the differentiation between bacterial and viral infection, absolute variable importance scores. Increase or decrease in the respective group is displayed on the right. e: M-fold cross validation of the mixOmics model (folds = 3). Differences between the overall classification error rate and the balanced error rate (BER) show a disbalance in the group sizes. f: Leave-one-out cross validation in MetaboAnalyst, good predictions will have a high R2 and Q2; a lower Q2 or a gap between R2 and Q2 of more than 0.3, means that the model is potentially overfitted.

Table S1 Descriptive statistics on the initial CAP cohort. Self-reported gender is given in male (M) and female (F). Age and the duration of the hospital stay are given in mean and standard deviation. ICU admission was counted, if a patient had to be treated at the ICU within at least one of the three explored visits. Moderate cases were defined as patients who were hospitalized but did not require a stay in the ICU or IMC. Severe cases were defined as patients who were hospitalized and required a stay in the ICU or IMC.

|  | **viral** | | **bacterial** | |
| --- | --- | --- | --- | --- |
| **N** | 43 | | 26 | |
| **Age [years]** | 53.3±17.1 | | 68.7±18.1 | |
| **Gender** | **Male** | 27 | **Male** | 17 |
|  | **Female** | 16 | **Female** | 9 |
| **Duration of hospital stay [days]** | 10.8±8.0 | | 13.4±8.6 | |
| **ICU admission** | **Yes** | 5 | **Yes** | 6 |
|  | **No** | 38 | **No** | 20 |
| **CAP severity** | **Moderate** | 35 | **Moderate** | 12 |
|  | **Severe** | 8 | **Severe** | 14 |

**Members of the CAPNETZ study network 2023 (with details of the institution)**

A. Fuchs^9^, M. Engelmann^9^, G. Paul^9^, M. Ayoub^9^, K. Groehl^9^, K. Riedl^9^, D. Stolz^10^, W. Bauer^11^, E. C. Diehl-Wiesenecker^11^, I. von Wunsch-Rolshoven Teruel^11^, N. Galtung^11^; N. Suttorp^12^, M. Witzenrath^12^, C. Wildberg^12^, C. Pley^12^, E. Zessin^12^, S. Schmager^13^, B. Schaaf^14^, J. Kremling^14^, D. Nickoleit-Bitzenberger^14^, H. Azzaui^14^, M. Hower^14^, F. Hempel^14^, K. Prebeg^14^, K. Popkirova^14^, M. Kolditz^15^, B. Schulte-Hubbert^15^, S. Langner^15^, G. Rohde^6^, C. Bellinghausen^6^, A. Grünewaldt^6^, A. Endres^6^, C. Frigerio^6^, B. Fiedler^6^, M. Panning^16^, T. Welte^17^, I. Pink^17^, N. Drick^17^, T. Fühner^18^, M. van’t Klooster^18^, T. Steinberg^18^, G. Barten-Neiner^19^, W. Kröner^19^, O. Unruh^19^, N. Adaskina^19^, F. Eberherdt^19^, C. Julius^19^, T. Illig^20^, N. Klopp^20^, M. Pletz^21^, B. T. Schleenvoigt^21^, C. Bahrs^21^, A. Moeser^21^, J. Ankert^21^, U. Sommerwerck^22^, T. Wintermantel^22^, D. Drömann^23^, P. Parschke^23^, K. Franzen^23^, J. Rupp^24^, F. Waldeck^24^, N. Käding^24^, C. Spinner^25^, J. Erber^25^, F. Voit^25^, J. Schneider^25^, M. Falcone^26^, G. Tiseo^26^, D. Heigener^27^, I. Hering^27^, W. Albrich^28^, F. Rassouli^28^, B. Wirth^28^, C. Neurohr^29^, A. Essig^30^, S. Stenger^309^, M. Wallner^31^, H. Burgmann^32^, L. Traby^32^, L. Schubert^32^, and all study nurses.

^6^Goethe University Frankfurt, University Hospital, Medical Clinic I, Department of Respiratory Medicine, Frankfurt/Main, Germany

^9^III. Medical Clinic, University Hospital Augsburg

^10^Clinic of Pneumology, University Hospital Basel, Switzerland / Clinic of Pneumology, University Hospital Freiburg

^11^Central Emergency Admission / Medical Admission Ward, Charité-Universitätsmedizin Berlin

^12^Clinic of Pneumology, Respiratory Medicine and Intensive Care Medicine with Study Area of Sleep Medicine, Charité-Universitätsmedizin Berlin

^13^Pneumology Section of II. Medical Clinic, Carl-Thiem Hospital Cottbus

^14^Pneumology, Infectiology and Internal Intensive Care Medicine, Medical Clinic Nord, Dortmund

^15^Medical Clinic I / Department of Pneumology, University Hospital Dresden

^16^Institute of Virology, University Hospital Freiburg

^17^Department of Pneumology, Hannover Medical School, Hannover

^18^Clinic of Pneumology, Intensive Care and Sleep Medicine, Siloah Hospital, Hannover

^19^CAPNETZ Office, Hannover

^20^Hannover Unified Biobank, Hannover Medical School

^21^Institute for Infection Medicine and Hospital Hygiene (IIMK), University Hospital Jena

^22^Cellitinnen-Severinsklösterchen Augustinerinnen Hospital, Cologne

^23^Medical Clinic III, Pneumology, University Medical Center Schleswig-Holstein, Lübeck

^24^Clinic of Infectious Diseases and Microbiology, University Hospital Schleswig-Holstein, Lübeck

^25^Clinic of Internal Medicine II, Infectiology, University Hospital rechts der Isar, Technical University of Munich

^26^Department of Clinical and Experimental Medicine, Università di Pisa

^27^Department of Pneumology, Agaplesion Diakonieklinikum Rotenburg

^28^Department of Infectiology and Hospital Hygiene, Kantonsspital St. Gallen, Switzerland

^29^Department of Pneumology and Respiratory Medicine, Robert Bosch Hospital, Stuttgart

^30^Institute of Medical Microbiology and Hygiene, University Hospital Ulm

^31^2mt Software, Ulm

^32^University Clinic of Internal Medicine I, Medical University of Vienna
